# Synergistic and off-target effects of bacteriocins in a simplified human intestinal microbiome: implications for *Clostridioides difficile* infection control

**DOI:** 10.1080/19490976.2025.2451081

**Published:** 2025-01-16

**Authors:** Natalia S. Ríos Colombo, R. Paul Ross, Colin Hill

**Affiliations:** a APC Microbiome Ireland, University College Cork, Cork, Ireland; b School of Microbiology, University College Cork, Cork, Ireland

**Keywords:** *C. difficile*, bacteriocin, microbiome, lantibiotic, pediocin PA-1, lacticin 3147, intestinal microbiome, SIHUMI

## Abstract

*Clostridioides difficile* is a major cause of nosocomial diarrhea. As current antibiotic treatment failures and recurrence of infections are highly frequent, alternative strategies are needed for the treatment of this disease. This study explores the use of bacteriocins, specifically lacticin 3147 and pediocin PA-1, which have reported inhibitory activity against *C. difficile*. We engineered *Lactococcus lactis* strains to produce these bacteriocins individually or in combination, aiming to enhance their activity against *C. difficile*. Our results show that lacticin 3147 and pediocin PA-1 display synergy, resulting in higher anti-*C. difficile* activity. We then evaluated the effects of these *L. lactis* strains in a Simplified Human Intestinal Microbiome (SIHUMI-C) model, a bacterial consortium of eight diverse human gut species that includes *C. difficile*. After introducing the bacteriocin-producing *L. lactis* strains into SIHUMI-C, samples were collected over 24 hours, and the genome copies of each species were assessed using qPCR. Contrary to expectations, the combined bacteriocins increased *C. difficile* levels in the consortium despite showing synergy against *C. difficile* in agar-based screening. This can be rationally explained by antagonistic inter-species interactions within SIHUMI-C, providing new insights into how broad-spectrum antimicrobials might fail to control targeted species in complex gut microbial communities. These findings highlight the need to mitigate off-target effects in complex gut microbiomes when developing bacteriocin-based therapies with potential clinical implications for infectious disease treatment.

## GRAPHICAL ABSTRACT

**Figure:**
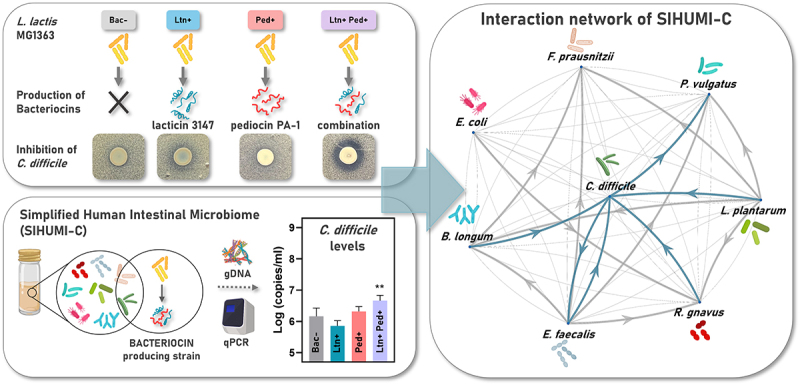

## 1. Introduction

*Clostridioides difficile* infection (CDI) is the leading cause of nosocomial diarrhea, predominantly affecting immunocompromised patients in hospitals, nursing homes, and elderly care facilities. *C. difficile* is an opportunistic pathogen, and most infections follow antibiotic therapy that causes disruptions to the gut microbiome.^1,2^ Over the past decade, there has been a notable increase in CDI outbreaks, presenting major challenges to healthcare facilities worldwide. Additionally, the severity and frequency of CDI cases have risen globally.^3^ *C. difficile* is also responsible for the majority (90–100%) of pseudomembranous colitis cases, a severe complication of antibiotic-associated diarrhea.^4^

Generally, the administration of an antibiotic is the first choice for CDI treatment in clinical practice and would usually involve either vancomycin, metronidazole or fidaxomicin.^5,6^ Vancomycin is effective against most *C. difficile* strains, including those with high levels of resistance to multiple antibiotics. However, its use can select for resistant strains of clostridia and other species, such as vancomycin-resistant enterococci, and may facilitate the spread of resistance determinants to other intra-hospital pathogenic bacteria.^7,8^ Consequently, metronidazole is usually the preferred drug.

Treatment failures and recurrence of CDI are highly frequent, with 15 to 30% of patients experiencing a relapse within three months after treatment with vancomycin and metronidazole.^1,9–11^ Since these are broad-spectrum antibiotics, it is suggested that they disrupt the gut microbiome, eradicating commensal bacteria and enabling the opportunistic re-growth of *C. difficile*. Consequently, these therapies not only fail to solve the problem but can also exacerbate it by perpetuating the microbiome disruptions that predispose patients to CDI.^12^ In fact, vancomycin and metronidazole are among the list of antibiotics known to facilitate CDI predisposition, along with antibiotics like clindamycin, ampicillin, amoxicillin, fluoroquinolones, and cephalosporins.^13–15^

Fidaxomicin is described as a narrow-spectrum antibiotic and represents an alternative treatment for CDI.^16,17^ It has shown similar clinical cure rates to vancomycin but with a reduced likelihood of recurrence.^2,18,19^ However, a recent study using a fecal fermentation model found that fidaxomicin affects numerous commensal gut bacteria and has a spectrum of activity comparable to broad-spectrum antibiotics.^20^

The reduced effectiveness of traditional treatments, the high frequency of patient relapses, and the rising prevalence of resistant hypervirulent strains of *C. difficile*^14,21^ highlight the threat of CDI and underscore the urgent need for new treatment strategies. Special attention is currently directed at non-conventional approaches, such as fecal microbiome transplants (FMT), probiotics and antimicrobial metabolites like bacteriocins.

FMT demonstrated effective results, preventing CDI recurrence in 80 to 90% of cases by restoring diversity and facilitating the engraftment of beneficial bacteria into the gut microbiome.^1,5,22,23^ However, CDI predominantly affects immunocompromised patients who frequently undergo prolonged antibiotic treatments and are often ineligible for FMT due to their weakened immune systems.

In this context, the use of probiotic strains and/or antimicrobial metabolites, such as bacteriocins, represents a promising alternative strategy for combating CDI.^24,25^ These approaches have the potential to preserve critical host gut microbiome functions – such as immune response modulation and pathogen colonization resistance – while reducing the risk of reinfection in immunodeficient patients.

Bacteriocins are antimicrobial peptides produced by various bacterial species, including many members of the human gut microbiome. They have been extensively studied as alternatives to antibiotics^26–28^ and as potential therapeutics for gastrointestinal diseases.^29,30^ In fact, commensal gut bacteria have been shown to provide colonization resistance to many pathogens and pathobionts through the production of bacteriocins. For example, a bacteriocin produced by the probiotic strain *Lactobacillus salivarius* UCC118 protects mice from *Listeria monocytogenes* infection,^31^ and bacteriocins from *Escherichia coli* Nissle 1917 limit gram-negative pathogens' growth during intestinal inflammation.^32^ Additionally, bacteriocins are notable for their low toxicity, their potential for *in situ* production by probiotic organisms, and their gene-encoded nature that allows for customization by bioengineering.^33^

When assessing bacteriocins for the treatment of CDI, delivery remains a considerable challenge. Orally-administered bacteriocins may be sensitive to proteolysis and may not survive gastric transit. In this regard, bacteriocin-producing strains offer an effective means of delivery in the highly proteolytic environment of the gut. Additionally, using bacteriocin-producing cultures in functional foods is more cost-effective and subject to fewer regulatory controls.^27,34^

Here, we engineered a set of *Lactococcus lactis* strains to produce two bacteriocins, lacticin 3147 and pediocin PA-1, that have different chemical structures, mechanisms of action and activity spectra that include pathogenic *C. difficile*.^35,36^ Lacticin 3147, a class I bacteriocin, is a two-peptide lantibiotic that requires substantial post-translational modifications for its activity and exhibits broad-spectrum inhibition of gram-positive bacteria. It targets lipid II, an essential player in peptidoglycan biosynthesis, and also induces membrane pore formation.^37,38^ Lacticin 3147 has shown *in vitro* antimicrobial activity against a range of clinically relevant *C. difficile* isolates at concentrations comparable to those of vancomycin and metronidazole used for therapeutic purposes.^33,35^ Lacticin 3147 has proven effective in eliminating *C. difficile* in a model of the human distal colon microbiome. However, to achieve the same anti-*C. difficile* effect in this model, lacticin 3147 required a threefold higher concentration than vancomycin or metronidazole. This concentration can adversely affect populations of *Lactobacillus* and *Bifidobacterium* and reduce overall microbial diversity.^35,39^

In contrast, pediocin PA-1, a class II bacteriocin, is a single linear peptide that undergoes no post-translational modifications other than the cleavage of a leader peptide upon export and has a relatively narrow spectrum. It binds to the mannose phosphotransferase transport system (Man-PTS) in sensitive strains, inducing pore formation and leading to cell death.^40,41^ Pediocin PA-1 has been reported to synergistically enhance the activity of the antimicrobial peptide durancin 61A against *C. difficile*.^42^ Thus, pediocin PA-1 as well as other pediocin-like bacteriocins are promising candidates that could act as adjuvants enhancing the activity of other bacteriocins.

The complementary use of antimicrobials with different mechanisms of action is an effective strategy to prevent the emergence of resistant pathogens, as it is more challenging for bacteria to concurrently develop resistance to two antimicrobials targeting different biological processes. Co-expression of lacticin 3147 and pediocin PA-1 could provide significant advantages in this regard, while potentially leading to synergistic effects.

In this work, *L. lactis* strains producing either lacticin 3147 (Ltn+) or pediocin PA-1 (Ped+) were tested against the toxigenic *C. difficile* strain VPI 10463. An isogenic non-producer strain (Bac-) was included as a negative control. We also engineered the same *L. lactis* strain to produce both bacteriocins simultaneously (Ltn+Ped+) to determine if the peptides exhibit synergy against *C. difficile*. However, a major drawback is our limited knowledge of the effectiveness of bacteriocin production in complex microbial communities such as the gut microbiome. As previously highlighted, when assessing novel approaches (like bacteriocin-producing strains) for CDI treatment, their impact on the gut microbial community is an important feature, as disruptive effects on gut microbial populations can facilitate disease recurrence. Understanding the impact of bacteriocins in the context of microbial communities rather than against individual strains in pure culture is crucial to avoid unforeseen consequences on the overall structure and function of these communities. Bacteriocins may not display the same activity against specific strains under different ecological conditions and can exert significant off-target effects within the microbial community.^43–46^

Predicting bacteriocin impacts on complex microbial communities through direct and indirect effects is challenging. The study of the human microbiome is limited by its complexity and the inter-subject variations. Defined *in vitro* polymicrobial communities offer a valuable resource for generating reproducible data in a controlled experimental setting. We optimized the culture conditions for a Simplified Human Intestinal Microbiome (SIHUMI), a defined consortium of seven well-characterized, fully sequenced commensal species isolated from the human gut. These bacteria are culturable *in vitro*, and their individual growth within the consortium can be tracked by qPCR using specific primers. Additionally, SIHUMI forms a stable population in the intestines of gnotobiotic mice of different genetic backgrounds (129S6/SvEv and C57BL/6), providing a platform for developing humanized mouse models for further complementary *in vivo* studies.^46–48^

In this work, we included *C. difficile* VPI 10463 to create a consortium we named SIHUMI-C. Bacteriocin-producing *L. lactis* strains were introduced to SIHUMI-C under simulated anaerobic and gut temperature conditions and the genome copy number of each SIHUMI-C member was evaluated over time to assess the impact of production of either lacticin 3147, pediocin PA-1, or both, on all members of the consortium.

Our results show an unexpected outcome: the combined effect of both bacteriocins led to an increase in *C. difficile* levels in the consortium despite displaying higher inhibitory activity when tested individually. These effects were analyzed considering antagonistic inter-species interactions within the SIHUMI-C community, providing insights into the ecological mechanisms by which broad-spectrum antimicrobials may fail to control *C. difficile* in complex microbial communities found in the human gut.

## 2. Materials and methods

### 2.1. Construction of bacteriocin-producing strains

We transformed *Lactococcus lactis* MG1363 strain with different plasmids, to make them produce either Lacticin 3147 (Ltn+), Pediocin PA-1 (Ped+), a combination of both bacteriocins (Ltn+Ped+), or none (Bac-), ensuring an isogenic background.

We used two MG1363 strains: one with pMRC01, a lactococcal plasmid containing lacticin 3147 operon,^49^ and the other with pMRC01∆*αβ*, a plasmid version lacking the two core peptide genes, *ltnα*/*ltnβ*^50^ as an isogenic non-lacticin producer. Pediocin PA-1 expression was achieved through the plasmid pNZ44-*pedApedD*,^51^ which contains the gene expressing the core peptide (including the leader sequence) followed by *pedD*, a gene encoding the ABC transporter named PedD, which identifies and cleaves the leader sequence during export of pediocin to the extracellular media. These genes are regulated by the constitutive promoter p44. The pNZ44-*pedApedD* plasmid was electroporated into *L. lactis* MG1363 competent cells with either pMRC01∆*αβ* (to obtain the Ped+ strain) or pMRC01 (to obtain the Ltn+Ped+ strain) according to the Holo and Nes protocol.^52^ An empty pNZ44 plasmid was electroporated into the same competent *L. lactis* strains, constituting the Bac- and Ltn+ strains respectively.

Electroporated cells were incubated overnight in LYHBHI medium^53^ supplemented with 5 µg/ml of chloramphenicol (Sigma Aldrich, Arklow, Ireland) to select for pNZ44-*pedApedD* and pNZ44. Table 1 shows the resulting bacteriocin-producing and non-producing strains.

**Table 1.** CHL^r^: chloramphenicol resistance. ERY^r^: erythromycin resistance.

Bacterial strains		Bacteriocin produced	Harboured Plasmids	Antibiotic Resistance
*Lactococcus lactis* MG1363	Bac-	None	pMRC01∆*αβ* pNZ44	CHL^r^
	Ltn+	Lacticin 3147	pMRC01 pNZ44	
	Ped+	Pediocin PA-1	pMRC01∆*αβ* pNZ44-*pedApedD*	
	Ltn+Ped+	Lacticin 3147 Pediocin PA-1	pMRC01 pNZ44-*pedApedD*	
*Listeria innocua* DPC3572		None (Indicator)	pNZ44E	ERY^r^

Unless stated otherwise, *L. lactis* strains were routinely grown aerobically at 30°C, in either liquid or solid LYHBHI medium with 5 µg/ml of chloramphenicol. LYHBHI consists of brain – heart infusion medium supplemented with 0.5% yeast extract, 5 mg/l hemin, 1 mg/ml cellobiose, 1 mg/ml maltose, and 0.5 mg/ml cysteine.^53^ All the supplements were purchased from Sigma – Aldrich.

### 2.2. Evaluation of bacteriocin production and activity

To test if the transformed *L. lactis* strains were capable of producing the corresponding bacteriocins in the same culture condition of SIHUMI-C (LYHBHI medium, at 37°C in anaerobiosis), we performed a deferred antagonism assay against a suitable indicator organism. Lacticin 3147 and, especially, pediocin PA-1 display activity against *Listeria* species, thus, *L. innocua* DPC3572 was used as an indicator strain to test bacteriocin production. *L. innocua* allowed us to work under Biosafety Level 1 conditions and it has been widely used in our laboratory for screening both class I and class II bacteriocins.

On the other hand we used *C. difficile* VPI 10463, a toxigenic strain originally isolated from an abdominal wound,^54^ as an indicator to evaluate a potential synergistic anti-*C.difficile* effect when lacticin 3147 and pediocin PA-1 are co-expressed by the same strain.

To perform the deferred antagonism assay, overnight cultures of the *L. lactis* strains (bacteriocin-producing and non-producing) were prepared. A 5 µl aliquot of each culture was spotted onto LYHBHI agar plates and incubated anaerobically at 37°C for 18 h. The plates were then exposed to UV light for 30 min. Indicator strains (*L. innocua* and *C. difficile*) were also grown overnight, after which 50 µl of each culture was inoculated into 10 ml of LYHBHI medium containing 0.75% agar. This was then overlaid onto the plates containing the *L. lactis* spots. The overlaid plates were incubated overnight at 30°C in aerobic conditions for *L. innocua* DPC3572 and at 37°C in anaerobic conditions for *C. difficile* VPI 10463. Bacteriocin production was confirmed by the presence of inhibition halos around the *L. lactis* spots, which were absent in the non-producing control. Synergistic effects between bacteriocins were indicated by an increase in the size of the inhibition halos compared to single bacteriocin production. This experiment was performed in triplicate.

### 2.3. Growth conditions of SIHUMI-C consortium

The SIHUMI consortium used in this study consists of fully sequenced human-derived intestinal bacteria: *Escherichia coli* LF82, *Enterococcus faecalis* OG1RF, *Lactiplantibacillus plantarum* WCFS1, *Faecalibacterium prausnitzii* A2–165, *Bifidobacterium longum* ATCC 15707, *Phocaeicola vulgatus* DSM1447 and *Ruminococcus gnavus* ATCC 29149.^48^ In our previous work, we found that LYHBHI medium^53^ is the best option for proper growth of all seven strains, including the fastidious anaerobes.^46^

In this work, we used a modified version, named SIHUMI-C, which includes an eighth member: *C. difficile* strain VPI 10463 (ATCC 43255),^54^ a strain that efficiently grows in the same culture conditions as the other SIHUMI members. All strains were grown in solid and liquid LYHBHI medium at 37°C in strict anaerobic conditions and were maintained as single-use glycerol stocks at −80°C for long periods.

### 2.4. Evaluation of inter-species interactions

Interactions among *C. difficile* and other members of SIHUMI-C were assessed by a cross-streaking method. A first streak of an overnight liquid culture of *C. difficile* was horizontally applied across an LYHBHI agar plate. A second streak of an overnight liquid culture of each of the other SIHUMI-C members was then perpendicularly applied, obtaining seven pairwise combinations. Plates were incubated for 48 h before assessing the results (this assay was performed in triplicate). Antagonism between two strains was considered positive when growth inhibition was observed, in either the first or the second streak.

### 2.5. Evaluation of the impact of bacteriocin-producing *L.* *lactis* on SIHUMI-C

We followed the protocol described in our previous work.^46^ Briefly, each SIHUMI-C strain was individually grown for 24 h in 5 ml of LYHBHI at 37°C under strict anaerobic conditions. The optical density at 600 nm (OD_600_) of each culture was measured by spectrophotometry and cultures were diluted with LYHBHI to get a final OD_600_ of 1. Ten µl of each culture was used to inoculate four tubes containing 10 ml of LYHBHI, forming four initial SIHUMI-C consortia. Simultaneously, overnight cultures of the four *L. lactis* strains (Bac-, Ltn+, Ped+ and Ltn+Ped+) were standardized to a final OD_600_ of 1, and 10 µl of each suspension was added to the different SIHUMI-C-containing tubes at time 0 h.

One ml samples were taken at 0, 6, and 24 h after inoculation, and centrifuged at 7000 rpm for 2 min. Supernatants were separated from the cell pellets, and both were stored at −20°C until processing.

The antimicrobial activity of SIHUMI-C supernatants was tested by spot assay against *L. innocua* DPC3572 transformed with the pNZ44-E plasmid,^46^ which allows erythromycin addition to prevent growth of residual bacteria in the supernatant. Sloppy LYHBHI medium (0.75% agar) supplemented with 5 µg/ml of erythromycin (Sigma Aldrich, Arklow, Ireland) was inoculated with 50 µl of an overnight culture of *L. innocua* and poured onto plates. Then, 10 µl of thawed supernatants were spotted and the plates were incubated aerobically at 30°C overnight.

Bacterial cell pellets were used for extraction of total genomic DNA (gDNA) using the GenElute Bacterial Genomic DNA Kit (Sigma Aldrich, Arklow, Ireland) following the manufacturer’s instructions and using a final elution buffer volume of 200 µl per sample.

### 2.6. Quantitative real-time PCR

The genome copy number of each species within SIHUMI-C at the different time points was determined from the total gDNA extracted, by qPCR using specific primers (Table 2). This allowed us to assess how the population of each strain in the consortium changed over time upon the addition of the bacteriocin-producing *L. lactis*.

**Table 2.** Selective species-specific primers for SIHUMI-C member strains and *L.* *lactis* MG1363. SIHUMI-C: Simplified Human Intestinal Microbiome containing *C. difficile*.

Target strain	Genome size (bp)	Genome Mass (ng)	Oligonucleotide Sequence (5’ to 3’)	Amplicon size	Reference
*E. faecalis* OG1RF	2739625	3.00 × 10^−6^	F: ACGGAGATTGTCACGCTTAGT R: TCGGCATTATCTGGGTGGTC	122 bp	^47^
*E. coli* LF82	4773108	5.23 × 10^−6^	F: CGGGTGTTGTCCTAACTGCT R: CGAGTGGTCATTGGCCTCAT	107 bp	^47^
*R. gnavus* ATCC 29149	3549191	3.89 × 10^−6^	F: GCGTGCTTGTATTCCGGATG R: GCCTGAACAGTTGCTTTCGG	115 bp	^75^
*F. prausnitzii* A2–165	3102523	3.40 × 10^−6^	F: TATTGCACAATGGGGGAAAC R:CAACAGGAGTTTACAATCCGAAG	77 bp	^76^
*P. vulgatus* DSM1447	4773108	5.23 × 10^−6^	F: AAGCAGCAGGGAAATGTGGA R: CTTTCCTTACTTGCGCGTCG	142 bp	^47^
*L. plantarum* WCFS1	3308274	3.63 × 10^−6^	F: CGAAGAAGTGCATCGGAAAC R: TCACCGCTACACATGGAGTT	71 bp	^76^
*B. longum* ATCC 15707	2385164	2.61 × 10^−6^	F: GAGGCGATGGTCTGGAAGTT R: CCACATCGCCGAGAAGATTC	108 bp	^77^
*C. difficile* VPI 10463	4313754	4.73 × 10^−6^	F: AAGCAGTAACAGTAGCAGTAGAA R: ATTTTCCAACTTCTTCATCACCA	114 bp	^55^
*L. lactis* MG1363	2529478	2.77 × 10^−6^	F: GCGATGAAGATTGGTGCTTGC R: ATCATCTTTGAGTGATGCAATTGC	173 bp	^46^

We have previously reported an accurate specificity for the set of primers targeting SIHUMI members.^46^ For the detection of *C. difficile* within SIHUMI-C, we used the specific primers designed by Kohler et al.,^55^ which target the Chaperonin-60 gene. Primer specificity for *C. difficile* was evaluated by PCR using as a template the genome of *C. difficile*, as well as all the other members of SIHUMI-C, to rule out a potential cross-amplification.

We used the qPCR protocol outlined in our prior study^46^ in the QuantStudio™ 5 Real-Time PCR instrument (Applied Biosystems, Thermo Fisher Scientific). Twenty μl reactions were set up in 96-well plates containing 1 × SYBR ChamQ Universal qPCR Master Mix (Generon), 0.4 μM of each primers, and 1 µl of extracted gDNA. The thermocycling protocol included a first step at 95°C for 2 min, and 45 cycles of 95°C for 15 s, 60°C for 15 s, and 72°C for 15 s. A negative control (no template) was included in every assay plate.

To calculate the genome copies/µl of each species within SIHUMI-C, we used standard curves that were prepared as described in our previous work.^46^ In brief, the genome masses were calculated by multiplying the genome size (bp) by the average molecular mass of a single bp in ng (1.096 × 10^−12^) (Table 2). gDNA was extracted from individual strains, and its concentration in ng/µl was used to calculate the necessary volumes to prepare a 3 × 10^6^ genome copies/µl solution. Tenfold (1:10) serial dilutions were then prepared to obtain solutions down to 3 copies/µl. Cycle threshold (Ct) values of each dilution were plotted against the known number of copies/µl of each dilution to create standard curves for each strain. Genome copy numbers in the mixed SIHUMI-C culture were calculated based on the measured Ct values of samples taken at each time point and expressed as copies/µl. The number of copies/ml was estimated by multiplying copies/µl by 200 (the final volume of gDNA eluted from 1 ml of culture sample). For more detailed information on standard curves preparation for SIHUMI, refer to our previous publication and the Applied Biosystems guidelines.^46,56^

### 2.7. Statistical analysis

Statistical analysis was conducted on four experimental replicates. The log of the genome copies for each SIHUMI-C member at 24 h was analyzed following the addition of Bac-, Ltn+, Ped+ or Ltn+Ped+ *L. lactis* strains to the consortium. Data normality was assessed using the D’Agostino-Pearson normality test in GraphPad Prism (v8). One-way ANOVA with Dunnett’s test was used to compare the mean genome copies of each SIHUMI-C member upon addition of bacteriocin-producing *L. lactis* (Ltn+, Ped+ and Ltn+Ped+) against the non-producing control (Bac-). Statistical significance was defined as *p* < 0.05.

## 3. Results

### 3.1. Lacticin 3147 and pediocin PA-1 display antimicrobial synergy when tested individually against *L.* *innocua* and *C.* *difficile*

**Figure 1.**
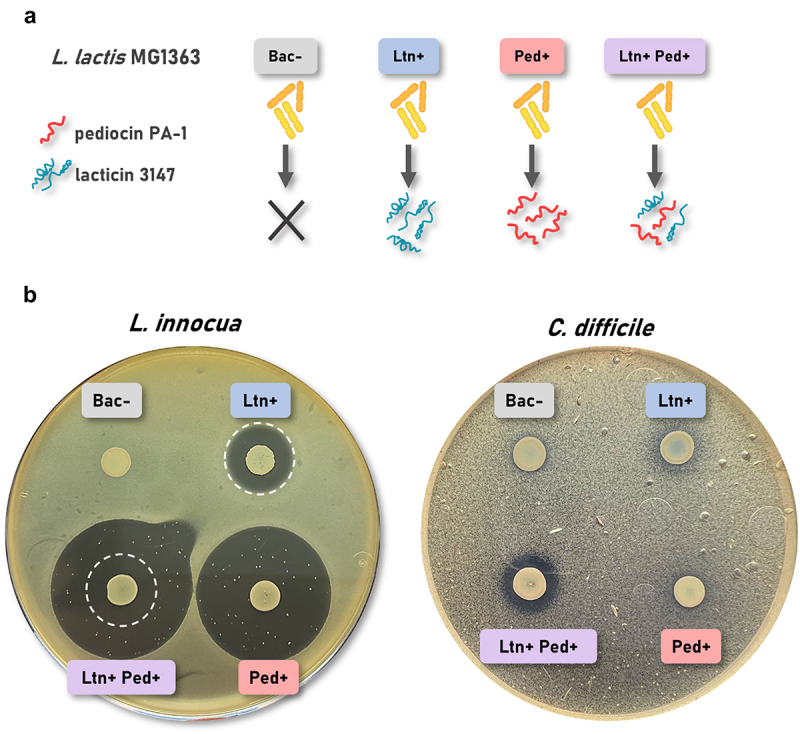
(a) Graphical depiction of the *L. lactis* MG1363 strains developed for this study. Bac-: non-producer, Ltn+: lacticin 3147-producer, Ped+: pediocin PA-1-producer, Ltn+Ped+: lacticin 3147 and pediocin PA-1 producer. (b) Antimicrobial activity of the *L. lactis* strains by deferred antagonism assay against *L. innocua* DPC3572 and *C. difficile* VPI 10463. The white dashed circles highlight areas with the same diameter, showing that the colony-free region of the Ltn+Ped+ halo matches the Ltn+ halo size.

We have previously shown that plasmids pMRC01 and pNZ44-*pedApedD* confer on *L. lactis* the ability to effectively express lacticin 3147 and pediocin PA-1, respectively. This was confirmed by inhibition halos displayed by the crude supernatants of the *L. lactis* strains against sensitive indicators and by MALDI-TOF mass spectrometry.^46,49,51^ Here we used the deferred antagonism assay to evaluate any potential antimicrobial synergy between lacticin 3147 and pediocin PA-1 when co-expressed by the same *L. lactis* host (Figure 1).

Production of lacticin 3147 and pediocin PA-1 was confirmed by the presence of halos of inhibition against *L. innocua* around spotted cultures of *L. lactis* Ltn+ and *L. lactis* Ped+. Though the antimicrobial activity was considerably more pronounced for pediocin PA-1, there are resistant colonies growing within the halo, a feature that was absent for lacticin 3147. Interestingly, co-expression of the two bacteriocins results in an inner halo free from colonies, that shares the same diameter as the Ltn+ halo (highlighted as white dashed circles in Figure 1b). This indicates that the Ltn+ Ped+ strain effectively produces both bacteriocins.

On the contrary, *C. difficile* only demonstrates slight sensitivity to lacticin 3147 as indicated by a hazy zone, while pediocin PA-1 gives no zone, similar to the non-producing control. However, co-expression of both bacteriocins generates a clear and bigger zone, suggesting an antimicrobial synergy against *C. difficile*. Therefore, the three producers were subsequently tested against the SIHUMI-C consortium.

### 3.2. *C.* *difficile* is inhibited by multiple members of SIHUMI-C consortium

Our previous work with the SIHUMI consortium demonstrated how inter-species interactions are major drivers for the final community structure and its response to antimicrobial agents such as bacteriocins. While for fecal microbiomes these interactions are usually unknown given its diversity, we showed that in a defined community like SIHUMI, the cross-streak method allows us to qualitatively assess antagonism between its members. Thus, all seven SIHUMI strains were cross-streaked against each other, and paired inhibitory interactions were identified to draft an antagonism network.^46^

**Figure 2.**
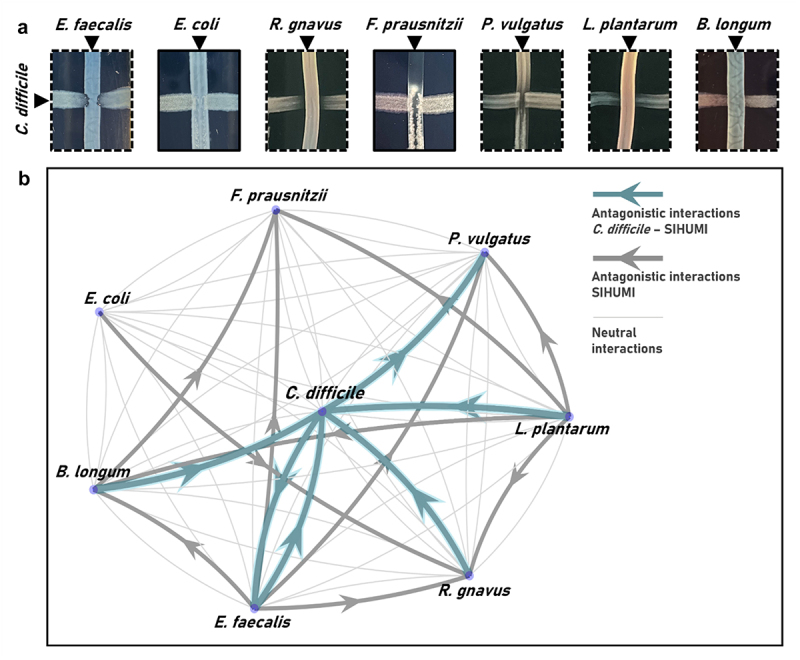
(a) Cross-streaking assay to assess antagonistic interactions between *C. difficile* and SIHUMI members. A first streak of *C. difficile* was horizontally applied across an LYHBHI agar plate. A second streak of each of the other SIHUMI-C members was then perpendicularly applied. Antagonism between two strains was considered positive when growth inhibition was observed, in either the first or the second streak (highlighted in dashed lines). (b) Interaction network diagram of SIHUMI-C consortium in solid LYHBHI. Each species is depicted as a node and interactions are depicted as edges connecting the nodes. Plain gray edges represent neutral interactions, weighted edges represent antagonistic interactions. The origin of the arrows indicates the antagonizing strain while the arrowheads point to the antagonized strain. Grey arrows depict antagonism identified in our previous work for SIHUMI^46^ while blue arrows represent the antagonism identified in this study between *C. difficile* and SIHUMI members.

SIHUMI-C incorporates *C. difficile* as a new member of this network; thus, antagonistic interactions between *C. difficile* and all seven SIHUMI strains were assessed using the cross-streak method (Figure 2a). Antagonism between two strains was considered positive when growth inhibition was observed in the streak of either species. Our results show that *C. difficile* is highly inhibited by multiple members of the consortium, including *E. faecalis, L. plantarum*, *R. gnavus* and *B. longum*. Interactions of *C. difficile* with *E. coli* and *F. prausnitzii* seem to be neutral, as no inhibition was apparent. On the other hand, *C. difficile* behaves as an inhibitor against *P. vulgatus*, and curiously, against *E. faecalis* as well. This bidirectional antagonism between *C. difficile* and *E. faecalis* was the only one of its kind identified for SIHUMI-C by the cross-streak assay method.

We then created an updated interaction network diagram for SIHUMI-C, where each species is depicted as a node and interactions are depicted as edges connecting the nodes (Figure 2b). Plain gray edges represent neutral interactions (when no antagonism was identified), and weighted edges represent antagonistic interactions. The origin of the arrows indicates the antagonizing strain while the arrowheads point to the antagonized strain. Grey arrows depict antagonism previously reported for SIHUMI^46^ while blue arrows represent the antagonism identified in this study between *C. difficile* and SIHUMI members.

In general terms, *E. faecalis* and *L. plantarum* are the main inhibitors (each inhibiting five other members of SIHUMI-C) while *R. gnavus*, *F. prausnitzii*, *P. vulgatus* and *C. difficile* are among the most inhibited by other members of the consortium. While we did not delve into the mechanisms behind each antagonism, the interaction network diagram of SIHUMI-C consortium largely explains the consortium behavior in response to the bacteriocin-producing *L. lactis* strains tested in this study.

### 3.3. Co-expression of lacticin 3147 and pediocin PA-1 by *L.* *lactis* increase levels of*C.* *difficile* when tested in the SIHUMI-C consortium

**Figure 3.**
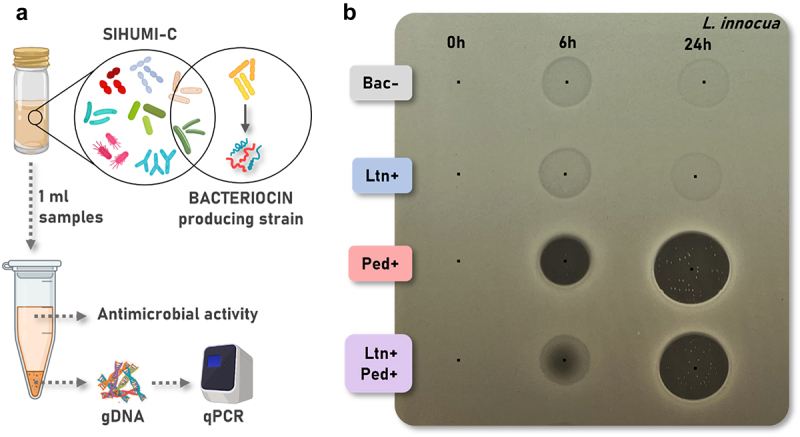
(a) Graphical depiction of the experimental procedure. SIHUMI-C consortium was inoculated in LYHBHI and the different bacteriocin producers and non-producer *L. lactis* strains were added at time 0 h. One ml samples were taken at 0, 6 and 24 h for centrifugation. Sample supernatants were used to evaluate bacteriocin production, while cell pellets were used for total genomic DNA (gDNA) extraction and quantitative real-time PCR (qPCR) analysis. Created with BioRender.com. (b) Antimicrobial activity by agar spot diffusion assays of SIHUMI-C supernatants at each time point against *L. innocua*. The black dots indicate where each supernatant was spotted. Bac- (non-producing control), Ltn+ (lacticin 3147), Ped+ (pediocin PA-1), Ltn+Ped+ (lacticin 3147 and pediocin PA-1).

We designed an experiment to assess the impact of either Bac-, Ltn+, Ped+ or Ltn+Ped+ *L. lactis* strains on the SIHUMI-C consortium (Figure 3a). The different bacteriocin-producing *L. lactis* strains were added to the SIHUMI-C consortium at time 0 h, and samples were taken at 0, 6 and 24 h for processing. Supernatants were used to evaluate bacteriocin production, while cell pellets were used for total genomic DNA (gDNA) extraction and qPCR analysis.

The antimicrobial activity of the SIHUMI-C supernatants was evaluated at each time point by spot assays against *L. innocua*, to determine if bacteriocins were effectively being produced in the context of the consortium (Figure 3b). When *L. lactis* Bac- is added, the consortium supernatants displayed hazy zones. This is almost certainly due to the inhibitory activity of *E. faecalis* against *L. innocua* reported previously.^46^ We also tested the anti-listerial activity of *C. difficile* supernatants (Supplementary Figure S1), as this is a new member in the consortium, and indeed, *C. difficile* displays some inhibitory activity against *L. innocua*, that might contribute to the halos observed when no bacteriocin is produced in the Bac- control.

Lacticin 3147 is not detectable in the supernatants of *L. lactis* Ltn+ samples (Figure 3b). This may be due to its lower intrinsic anti-listerial activity compared to pediocin PA-1 and its broad-spectrum nature, which means that the peptides would be expected to bind to all gram-positive strains in SIHUMI-C,^46^ including *C. difficile* (Figure 1b). A significant fraction of any lacticin 3147 that has been produced most likely remains in the cell pellet after centrifugation due to binding to target cells, rendering it undetectable in the supernatant. It should also be noted that spot diffusion assays provide only a rough estimation of actual antimicrobial bioavailability. On the other hand, pediocin PA-1 is successfully detected in the SIHUMI-C supernatant, as shown by the clear inhibitory zones against *L. innocua* at 6 and 24 h (Figure 3b).

However, when both bacteriocins are co-expressed, the supernatants display slightly smaller zone sizes than those of pediocin PA-1 alone. This suggests that the production levels of pediocin PA-1 are lower when co-expressed with lacticin 3147 compared to when it is expressed alone by the same *L. lactis* host.

Next, gDNA extracted from bacterial cell pellets was used to quantify the genome copies/ml of each SIHUMI-C member in the consortium by qPCR. Species-specific primers allowed for individual tracking of each member at different time points after addition of the *L. lactis* strains.

**Figure 4.**
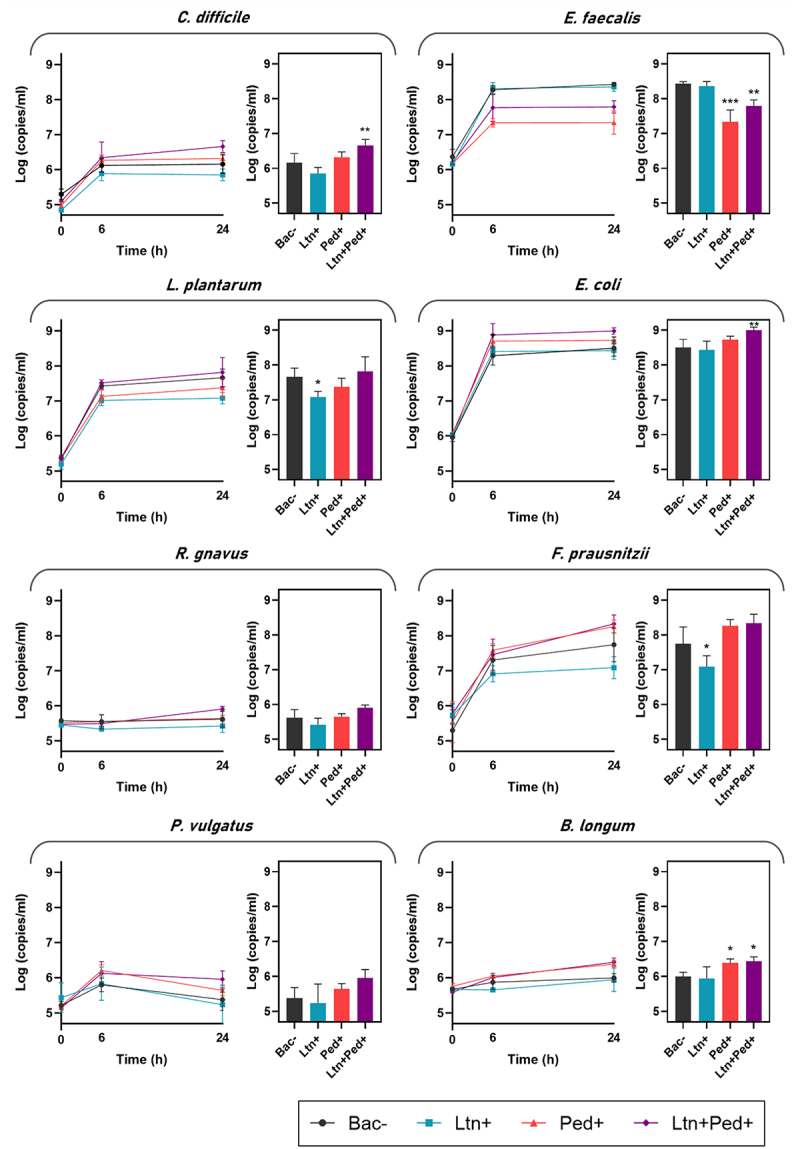
Log genome copies/ml over time (0, 6, and 24 h) of members of SIHUMI-C consortium in LYHBHI after inoculation with the bacteriocin producers and non-producer *L. lactis* strains at time 0 h. Each time point is represented as a mean with standard deviation of four replicates. Log genome copies/ml at 24 h after bacteriocin-producer treatment was compared to the non-producing control. Statistical significance was analyzed by comparing each treatment to the Bac- control and was recorded as follows: *** (*p* < 0.001), ** (*p* < 0.01), * (*p* < 0.05), no asterisk means no significant difference (*p* > 0.05). Bac- (non-producing control), Ltn+ (lacticin 3147), Ped+ (pediocin PA-1), Ltn+Ped+ (lacticin 3147 and pediocin PA-1).

We tracked the log genome copies/ml over time (0, 6, and 24 h) of members of SIHUMI-C consortium after inoculation with the bacteriocin producers and non-producer *L. lactis* strains at time 0 h (Figure 4). The mean of the log genome copies/ml at 24 h of each SIHUMI-C strain in the consortium after bacteriocin-producer treatment was compared to the non-producing control. It is worth highlighting that lacticin 3147 targets virtually all of the gram-positive strains within SIHUMI-C while pediocin PA-1 is very narrow-spectrum, displaying inhibition zones only against *E. faecalis*.^46^

Here, we present a comprehensive species-by-species analysis within the framework of a consortium of competing bacteria, examining the impacts of bacteriocins on both targeted and non-targeted members.

#### 3.3.1. C. difficile

As previously noted, *C. difficile* is inhibited by multiple members of SIHUMI-C, suggesting that the consortium plays a significant role in maintaining *C. difficile* at a relatively low level. While *L. lactis* producing lacticin 3147 slightly reduces *C. difficile* abundance, this difference was not statistically significant. Similarly, Pediocin PA-1 does not produce significant changes in *C. difficile* levels. However, the strain co-expressing both peptides led to a modest but significant increase in *C. difficile* levels in the consortium, an unexpected effect given the antimicrobial synergy between lacticin 3147 and pediocin PA-1 when individually tested against *C. difficile* (Figure 1b). This phenomenon could be explained by the fact that these bacteriocins also target other members of the consortium, leading to important off-target effects beyond their direct action on *C. difficile*, as shown by the interaction networks governing SIHUMI-C behavior. *C. difficile* is inhibited by *E. faecalis* (which is strongly targeted by pediocin and weakly by lacticin), *L. plantarum* and *B. longum* (which are targeted only by lacticin) (Figure 2).^46^ The resulting balance of interactions leads to an increase in *C. difficile* due to the reduction in the levels of these antagonistic bacteria. This underscores how the inhibitory potential of bacteriocins might be overestimated by conventional agar-based screening against single strains.

#### 3.3.2. E. faecalis

As shown in the interaction network (Figure 2b), *E. faecalis* is a potent antagonistic member within the community, so the direct inhibition of this strain causes indirect increases in multiple other members. This strain has been reported to be strongly inhibited by pediocin PA-1,^51^ and only slightly inhibited by lacticin 3147.^46^ This is further supported by the deferred antagonism assay against *E. faecalis* shown in Supplementary Figure S2, where Ltn+ *L. lactis* displays a very slight inhibition zone, while both Ped+ and Ltn+Ped+ strains show substantial inhibition zones. This aligns with the effect observed in the SIHUMI-C consortium (Figure 4), where lacticin 3147 has no significant impact on *E. faecalis* compared to the Bac- control strain, while pediocin PA-1 causes a consistent reduction in the levels of *E. faecalis*. Co-expression of both peptides clearly decreases *E. faecalis* numbers, though to a lesser extent as compared to the Ped+ treatment. This agrees with the decreased anti-listerial activity displayed by SIHUMI-C supernatants (Figure 3b) treated with Ltn+Ped+ *L. lactis* compared to Ped+ *L. lactis*. Antagonism between *E. faecalis* and *C. difficile* might explain why the Ltn+Ped+ strain causes an increase in levels of *C. difficile*, despite displaying an increased antimicrobial effect when individually tested against the same strain. Though the antagonism between *E. faecalis* and *C. difficile* is bidirectional, *E. faecalis* is among the most abundant members in the community, and it almost certainly plays a key role in limiting the growth of *C. difficile*. It is likely that the inhibition of *E. faecalis* facilitates *C. difficile* growth in the context of the consortium.

#### 3.3.3. L. plantarum

This strain acts as another potent inhibitor within the community, and it is significantly reduced by lacticin 3147, though it is not significantly impacted by pediocin PA-1 (Figure 4). In contrast, the co-expression of both peptides in the consortium has no significant effect on *L. plantarum*, suggesting that the levels of lacticin 3147 might be reduced when co-expressed with pediocin PA-1 compared to when it is expressed alone by the same *L. lactis* host. *L. plantarum* antagonizes *C. difficile* (Figure 2), and although there is no evident decrease in *L. plantarum* with Ltn+Ped+ *L. lactis*, lacticin 3147 might functionally affect the inhibitory effect of *L. plantarum* against antagonized members, resulting in a concomitant off-target increase of these, including *C. difficile*.

#### 3.3.4. E. coli

*E. coli* is a dominant member within the community and remains unaffected by Ltn+ or Ped+ strains compared to the Bac- controls (Figure 4), due to the lack of direct inhibitory effects exerted by these bacteriocins against gram-negative bacteria. However, the co-expression of lacticin 3147 and pediocin PA-1 significantly boosts *E. coli* levels in SIHUMI-C. The reason behind this increase is not fully clear, as *E. coli* barely interacts with other members of SIHUMI-C in one-to-one experiments. A possible explanation might be that the simultaneous drop of individual targeted species increases the availability of nutrients for *E. coli*, benefiting its growth.

#### 3.3.5. R. gnavus

This strain acts as a *C. difficile* inhibitor but it is strongly antagonized by other members (*E. faecalis, L. plantarum* and *E. coli*) which leads to the very low levels of this strain in the consortium (Figure 4). *R. gnavus* levels remain unaffected by the different bacteriocin-producing strains after 24 h.

#### 3.3.6. F. prausnitzii

This strain is strongly inhibited by *E. faecalis*, *B. longum* and *L. plantarum* (Figure 2). However, it reaches higher levels than *R. gnavus*, probably due to a faster growth rate or a higher carrying capacity within the community. Lacticin 3147 has reported activity against *F. prausnitzii*,^46^ which explains the direct impact that the Ltn+ *L. lactis* has on *F. prausnitzii* in SIHUMI-C (Figure 4), leading to a substantial reduction. Pediocin PA-1 seems to boost the levels of *F. prausnitzii*, though not significantly. The direct reduction of *E. faecalis* by pediocin PA-1 might result in an indirect benefit for *F. prausnitzii* in the consortium. Interestingly, the strain co-expressing both peptides fails to reduce *F. prausnitzii* levels. This might be due to a reduced production of lacticin 3147 when co-expressed with pediocin PA-1 (as seen with *L. plantarum*), as well as an indirect boost caused by the effect of pediocin PA-1 on *E. faecalis*.

#### 3.3.7. P. vulgatus

Because this strain is highly inhibited by other members in SIHUMI-C, *P. vulgatus* remains among the less abundant species, along with *R. gnavus*. Furthermore, akin to *E. coli*, *P. vulgatus* is a gram-negative species and thus remains largely unaffected by the tested bacteriocins.

#### 3.3.8. B. longum

Though lacticin 3147 has reported antimicrobial activity against *B. longum*,^46^ the growth of *B. longum* in the SIHUMI-C is not significantly impacted by lacticin 3147. Interestingly, *B. longum* benefits from the presence of both Ped+ and Ltn+Ped+ producers (Figure 4), probably due to an indirect consequence of inhibition of *E. faecalis*. These effects arise due to the strong antagonism exerted by *E. faecalis* upon *B longum*.

#### 3.3.9. L. lactis

The fate of the different *L. lactis* strains introduced to SIHUMI-C was also assessed by qPCR (Supplementary Figure S3). There were no statistically significant differences in the genome copies of any of the *L. lactis* strains in SIHUMI-C at 24 hours. It appears that bacteriocin production does not impact the behavior of the producer strains.

In general, a decreased antimicrobial effect against sensitive SIHUMI-C members is observed when both bacteriocins are co-expressed within the consortium. Pediocin PA-1 shows reduced antimicrobial activity against *E. faecalis* when co-expressed with lacticin 3147 compared to when expressed alone. Similarly, lacticin 3147 exhibits a decreased antimicrobial effect against *C. difficile*, *L. plantarum*, and *F. prausnitzii* when co-expressed with pediocin PA-1, compared to when it is expressed alone. This observation aligns with the bacteriocin bioavailability analysis of supernatant samples from SIHUMI-C (Figure 3b), where co-expression of both peptides resulted in reduced activity against *L. innocua* compared to the production of only pediocin by the same *L. lactis* host.

The use of the SIHUMI-C model, with its simplified composition, enables the detailed dissection of mechanisms through which bacteriocins affect microbial populations beyond *C. difficile*. These findings hold significant translational relevance, as they could contribute to the development of next-generation bacteriocin therapies by guiding the identification or design of bacteriocins with enhanced specificity and efficiency for modulating the gut microbiome.

## 4. Discussion

While the importance of conventional antibiotics cannot be overstated, their prolonged and sometimes inappropriate use can lead to negative consequences, including the disruption of protective and commensal microbiomes. There is a clear need for more selective and effective “smart” antibacterial agents that can precisely target *C. difficile*.

The existing literature suggests that bacteriocins, combinations of bacteriocins, and bioengineered variants may offer promising alternative approaches to combat CDI.^57^ However, the therapeutic application of bacteriocins in human medicine is a relatively new area of research. The inhibitory effects of bacteriocins are typically tested on a limited set of individual strains in pure culture, which almost certainly does not accurately represent their likely impact on complex and dynamic communities such as those found in the gut microbiome. Consequently, it remains uncertain how bacteriocin production in the gut might affect the overall composition and function of the microbiome.

In this work, we engineered a set of *L. lactis* strains to express two bacteriocins from different classes and with different mechanisms of action, lacticin 3147 and pediocin PA-1, both of which have reported activity against *C. difficile*. However, their effects when produced by *L. lactis* strains have not been studied.

*L*. *lactis* MG1363 has GRAS (Generally Regarded as Safe) status and due to its long history of safe use and the availability of advanced genetic engineering tools, it is highly versatile and well-suited for biotechnological applications. Although not a natural resident of the human gastrointestinal tract, it can survive passage through the gut and has been successfully used to deliver bioactive peptides.^58,59^ This makes it a promising candidate for delivering bacteriocins during the proteolytic digestive transit.

To our knowledge, only limited studies have evaluated the combined applications of bacteriocins for synergistic antimicrobial action against *C. difficile*.^42,60^ Therefore, we assessed the combined action of lacticin 3147 and pediocin PA-1 produced by the same *L. lactis* host. We evaluated potential synergy against *C. difficile* using deferred antagonism and within the context of a human gut-derived community named SIHUMI-C, under simulated anaerobic and temperature conditions of the gut.

Our results show an unexpected outcome in that the combined effect of both bacteriocins increases *C. difficile* levels in the consortium, despite displaying higher inhibitory activity against the same strain when tested individually by the deferred antagonism assay. We propose that this phenomenon can be explained by the inter-species interaction network outlined in this study. Inter-species interactions are major determinants of community assembly^61–63^ as species compete for limited resources like nutrients and space. While such interactions in complex communities like microbiomes are often unknown due to their scale and intricacy,^64^ a simplified consortium like SIHUMI-C allows for the dissection of these interactions. We previously found that the cross-streak method can rapidly provide qualitative insights into antagonisms among bacterial strains.^46^ Given that SIHUMI-C includes *C. difficile*, we generated an updated interaction network indicating the various antagonistic interactions among all eight SIHUMI-C strains. This network helps explain the changes observed in response to the tested bacteriocins. Strains like *E. faecalis* and *L. plantarum* act as principal controllers of population composition, inhibiting multiple members within the consortium. While the specific inhibitory mechanisms were not the primary focus of this research, it is plausible that bacteriocin production plays a significant role, as it is a highly common strategy for competition and survival in the gut ecosystem. Using BAGEL4, a bioinformatics tool for identifying bacteriocin biosynthetic gene clusters,^65^ we identified that *L. plantarum* may be highly bacteriocinogenic, encoding five different plantaricins (K, J, N, A, and F). This could explain the strong antagonistic activity observed for *L. plantarum*. On the other hand, *E. faecalis* does not seem to encode bacteriocin operons based on genome analysis. However, it does retain several core virulence factors and other competition-related traits that could allow it to inhibit other species, particularly under conditions of dysbiosis.^66^ Further investigation into these mechanisms would be valuable for understanding SIHUMI-C dynamics.

On the other hand, strains like *R. gnavus*, *P. vulgatus*, and even *C. difficile* show limited growth, suggesting the consortium plays a key role in controlling *C. difficile* overgrowth. Since pediocin PA-1 has potent activity against *E. faecalis*, and lacticin 3147 targets *L. plantarum* and other SIHUMI-C members,^46^ their co-expression removes the antagonistic pressure on *C. difficile*, facilitating its growth. Our results reinforce our previous conclusions: SIHUMI-C behaves such that specific strain-directed knockdowns by different bacteriocins generate a “domino effect,” leading to broader consequences in other members due to the inter-species interaction network.

It is clear that the inhibitory potential of bacteriocins might be wrongly predicted by conventional agar-based screening against single strains. While lacticin 3147 and pediocin PA-1 display synergy on agar-based tests against sensitive strains like *L. innocua* and *C. difficile*, their co-expression by the same host in a polymicrobial community context diminishes their overall antimicrobial efficiency against specific target species, as shown for *E. faecalis*, *L. plantarum*, and *F. prausnitzii*. Furthermore, it can indirectly boost the growth of others, such as *E. coli* and *B. longum* and even benefit opportunistic pathogens like *C. difficile*, despite the combined bacteriocins being inhibitory against that target. The impact of bacteriocins extends beyond the targeted knockdown of sensitive species within a bacterial consortium, and individual tests cannot easily predict how antimicrobials will behave in a community. Thus, testing efficacy within community contexts like SIHUMI-C is key to addressing potential species-targeted interventions in the gut microbiome.

Multiple studies, including those presented in this work, underscore the importance of specificity alongside potency against *C. difficile* to prevent off-target effects that disrupt the gut community.^67–69^ Efforts should prioritize testing bacteriocins with high potency and, equally important, specificity against *C. difficile* to maintain microbial community balance, crucial for controlling *C. difficile* outbreaks.

In this regard, Thuricin CD has proven to be an excellent candidate. Thuricin CD is a narrow-spectrum bacteriocin produced by *Bacillus thuringiensis* DPC 6431, with potent activity against a wide range of clinical *C. difficile* isolates and minimal impact on other gut microorganisms.^33^ Studies have demonstrated the effectiveness of Thuricin CD in killing *C. difficile* in a distal colon model, comparable to traditional antibiotics vancomycin and metronidazole, without significantly altering the microbiome composition.^39^ Recent research has compared Thuricin CD with fidaxomicin, revealing Thuricin CD to be superior in terms of specificity and minimizing collateral damage to the gut microbiome, maintaining efficacy in completely eliminating *C. difficile*.^20^ Future studies should test Thuricin CD-producing and non-producing strains in the SIHUMI-C model to evaluate whether its specificity offers better outcomes against *C. difficile* compared to the bacteriocins evaluated in this study. This could pave the way for more precise therapeutic strategies to effectively control *C. difficile* while minimizing disruption to the commensal gut microbiota.

We acknowledge the need for further research to understand how the inhibition of key commensal species affects bacteriocin-targeted interventions for CDI, potentially creating niches that favor *C. difficile* proliferation.^70,71^ This increase in *C. difficile* may also result from compensatory overexpression of nutrient acquisition pathways, enabling it to thrive in environments vacated by sensitive species.^72,73^ Investigating bacteriocin-specific resistance mechanisms and their impact on *C. difficile* gene expression could provide valuable insights. Moreover, exploring alternative delivery systems for bacteriocins – such as encapsulation or pairing with microbiome-stabilizing agents like prebiotics^74^—could help minimize disruptions and enhance therapeutic outcomes for recurrent *C. difficile* infections.

## 5. Conclusion

Seeking therapeutic alternatives to conventional antibiotics for treating CDI poses a significant challenge. In this context, bacteriocins stand out as a highly promising alternative. Our work addresses crucial aspects such as delivery systems, antimicrobial synergy, and ecological impacts within the gut microbiome, thereby advancing the understanding of bacteriocins’ therapeutic potential. We underscore the importance of considering interaction networks within gut microbial communities when developing targeted interventions. Additionally, we highlight the value of simplified systems like SIHUMI-C, which mitigate inter-individual variation that can occur in studies using human fecal microbiomes. While our findings provide valuable and reproducible insights, further research is necessary to translate these results into clinical applications and support the rational design of novel bacteriocins or their combination with other existing therapies. Continued exploration of bacteriocins is imperative to develop safer and more effective treatments for CDI and beyond.

## Supplementary Material

Supplementary material.docx

## Data Availability

The authors declare that all the data supporting our findings in the study are available within the paper.
