# Supplementary material for: Synergistic and off-target effects of bacteriocins in a simplified human intestinal microbiome: implications for Clostridioides difficile infection control
Source: Gut Microbes. 2025 Jan 16;17(1):2451081. doi: 10.1080/19490976.2025.2451081 (PMC11740676; doi:10.1080/19490976.2025.2451081)
Supplement: Supplementary material.docx [file KGMI_A_2451081_SM9387.docx]

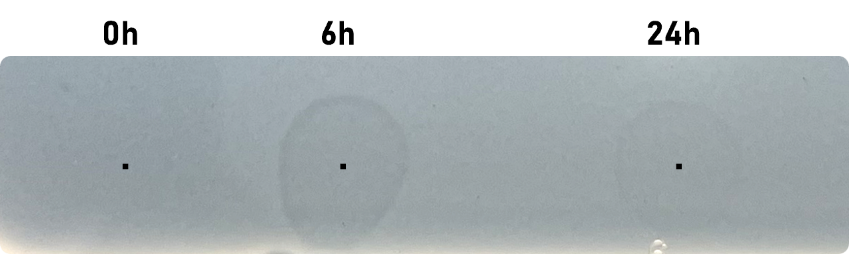


**Supplementary Figure 1:** Antimicrobial activity of *C. difficile* culture supernatants by agar spot diffusion method against *L. innocua*. The black dots indicate where each supernatant was spotted.


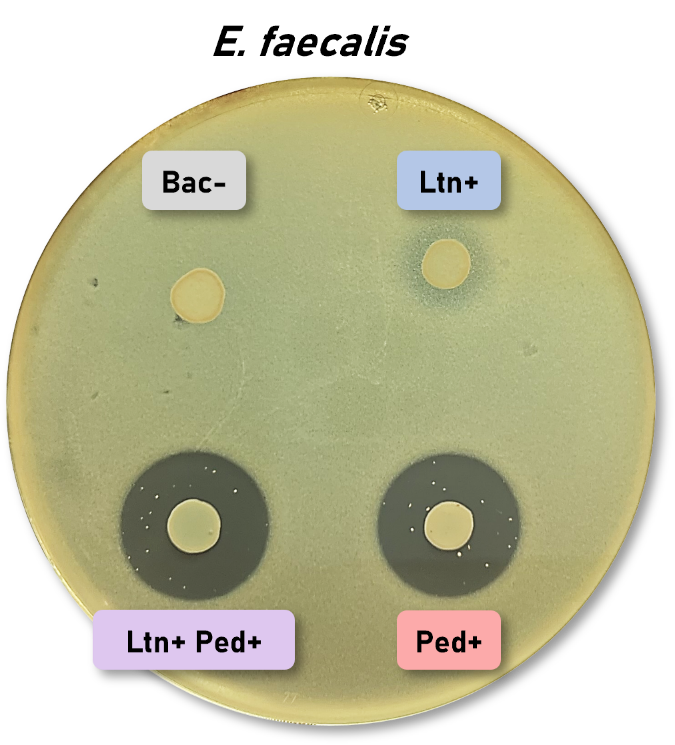


**Supplementary Figure 2:** Antimicrobial activity of the *L. lactis* strains by deferred antagonism assay against *E. faecalis* OG1RF, a member of SIHUMI-C. Lacticin 3147 displays a very slight inhibition zone, while both pediocin PA-1 and the combination of both peptides show substantial inhibition zones. Bac-: non-producer, Ltn+: lacticin 3147-producer, Ped+: pediocin PA-1-producer, Ltn+Ped+: lacticin 3147 and pediocin PA-1 producer.


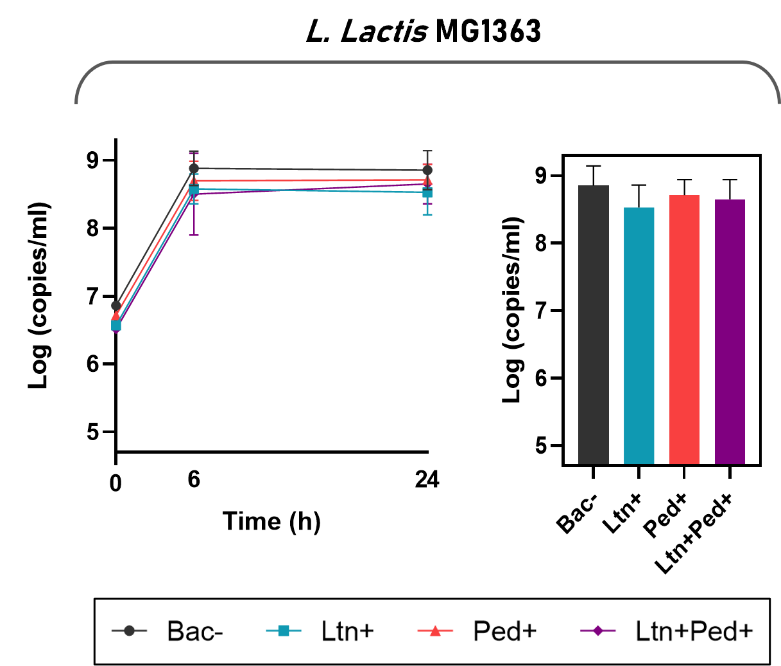


**Supplementary Figure 3:** Log genome copies/ml over time (0, 6, and 24 h) of bacteriocin-producing and non-producing *L. lactis* strains growing within the SIHUMI-C consortium in LYHBHI. Each time point is represented as a mean with standard deviation of four replicates. Log genome copies/ml at 24 h was compared to the non-producing control resulting in no statistical significance (p> 0.05). Bac- (non-producing control), Ltn+ (lacticin 3147), Ped+ (pediocin PA-1), Ltn+Ped+ (lacticin 3147 and pediocin PA-1).
